# External Error Monitoring in Subclinical Obsessive-Compulsive Subjects: Electrophysiological Evidence from a Gambling Task

**DOI:** 10.1371/journal.pone.0090874

**Published:** 2014-03-07

**Authors:** Chunyan Zhu, Fengqiong Yu, Rong Ye, Xingui Chen, Yi Dong, Dan Li, Long Zhang, Dandan Li, Kai Wang

**Affiliations:** 1 Laboratory of Cognitive Neuropsychology, Department of Medical Psychology, Anhui Medical University, Hefei, China; 2 Department of Neurology, The First Affiliated Hospital of Anhui Medical University, Hefei, China; 3 Anhui Mental Health Center, Hefei, China; Bellvitge Biomedical Research Institute-IDIBELL, Spain

## Abstract

**Background:**

Feedback-related negativity (FRN) is believed to be an important electrophysiology index of “external” negative feedback processing. Previous studies on FRN in obsessive-compulsive (OC) individuals are scarce and controversial. In these studies, anxiety symptoms were not evaluated in detail. However, OC disorders have a number of radical differences from anxiety disorders. It is necessary to study FRN and its neuroanatomical correlates in OC individuals without anxious symptoms.

**Methods:**

A total of 628 undergraduate students completed an OC questionnaire. We chose 14 students who scored in the upper 10% and 14 students who scored in the lowest 10% without anxiety symptoms as a subclinical OC group (SOC) and a low obsessive-compulsive group (LOC). The students all performed the revised Iowa Gambling Task. We used the event-related potentials (ERP) and standardized low-resolution brain electromagnetic tomography (sLORETA) to track external negative feedback processing and its substrate in the brain.

**Results:**

Our study revealed poorer decision-making ability and greater FRN amplitudes in SOC subjects compared with LOC controls. The SOC subjects displayed anterior prefrontal cortex (aPFC) hyperactivation during the loss feedback condition. Specifically, we found an intercorrelation of current source density during the loss condition between the dorsal anterior cingulate cortex (dACC) and aPFC in the LOC subjects but not in the SOC group.

**Conclusions:**

Our results support the notion that overactive external feedback error processing may reflect a candidate endophenotype of OC. We also provide important information on the dysfunction in the interaction between aPFC and dACC in populations with OC. Nevertheless, the findings support that OC may be distinguished from other anxiety disorders using a new electrophysiology perspective.

## Introduction

Obsessive-compulsive (OC) symptoms are characterized by recurrent intrusive thoughts (obsessions) and repetitive behaviors or mental acts (compulsions), which are time-consuming and lead to significant functional impairments or related anxiety. These symptoms occur not only in obsessive-compulsive disorders (OCD) but are also found in general populations with subclinical obsessive-compulsive (SOC) symptoms, which are considered to be “traits and symptoms of OCD that are not severe enough to meet OCD criteria” [1]. In the past decade, investigations using electrophysiological (event-related potentials, ERP) and event-related functional magnetic resonance imaging (fMRI) have converged to implicate a dysfunctional “error monitoring system” in the etiology of obsessive-compulsive (OC) symptoms [2]–[4]. The involvement of the prefrontal cortex (PFC) [5], the anterior cingulate cortex (ACC) [2], [6] and the corpus striatum [7], [8] in traditional cortico-striato-thalamo-cortical (CSTC) circuits may be related to OC symptoms, such as abnormal error processing.

Previous studies on the error monitoring function in OCD and SOC focused mainly on excessive and persistent error-related brain activity [9]–[11] and error-related brain potentials (error-related negative wave, ERN) [4], [12]. In these previous studies, when an impulsive error was committed in speeded reaction time tasks, such as the flanker task or the go-nogo task [13], [14], the subjects would self-detect the error at the same time. The sources of error information include not only “internal” signals generated from an “efference copy” of the response command, but the fact that the individual decision-making process relies equally on “external” feedback signals to determine whether the responses are correct. The processing of negative performance feedback is a critical component in decision-making [15] that allows for flexible adjustment to optimize behavioral outcomes. Learning from external negative feedback is a hallmark of human social and emotional development, which may lead to a different psychological and physiological state in the decision-maker [16]. Therefore, studying “external” negative feedback processing and its neuroanatomical correlates in OC individuals might be a fruitful approach for further clarifying the influence of error monitoring in the pathophysiology of related diseases.

ERP is a tool with high temporal resolution that offers the opportunity to track external feedback error monitoring and its substrate in the brain. A negative brain potential labeled feedback-related negativity (FRN) is believed to be an important index of feedback processing. FRN is a negative deflection that peaks approximately 200–300 ms after the presentation of feedback in a time-estimation task [17], a probabilistic learning task [18] and especially in gambling tasks [19], [20]. Studies have demonstrated that the FRN has a pronounced sensitivity to the valence of the feedback [11], [19], [20], unexpected outcomes [14], [21] and response conflict [14]. Holroyd and Coles (2002) suggested that the FRN is the ERP component of reward prediction error feedback processing. Several sources of evidence suggest that the FRN reflects the principles of reinforcement learning which is moderated by dopamine [18]. EEG source localization and EEG-informed fMRI have both strongly implied that the FRN is generated from the dorsal anterior cingulate cortex (dACC) [10], [20], [22]. Some ERP studies reported aberrant FRN in neurological and psychiatric diseases, such as gambling [23], alcoholism [24], schizophrenia [25] and attention-deficit hyperactivity disorder (ADHD) [26].

Several lines of evidence have suggested that subjects with OC symptoms [27] exhibit overactive internal error monitoring with increased amplitude of the ERN and increases in PFC and ACC activation. Based on the resemblances between the FRN and ERN, and their seemingly similar circumstances of occurrence [28], it stands to reason that the FRN may also be enhanced in OCD patients. However, current FRN studies on external feedback error monitoring in OCD are scarce and controversial. In addition to a trend for larger FRN found in OCD patients in one study [29], reduced FRN amplitudes have been reported in limited studies. Contrary to their prediction hypothesis that the increasing FRN was expected to find in High OC subjects by Simons, High OC subjects had smaller FRN [30]. According to the results of Endrass, OCD patients also displayed reduced FRN amplitudes for exploration negative feedback [31]. O’Toole’s et al. reported that high OC individuals demonstrated aberrant feedback monitoring as characterized by a lack of differentiation to the valence of feedback [32]. In these studies, anxiety symptoms were not evaluated and described in detail. In some of these studies, OCD was even identified as one type of chronic anxiety disorder [30]. Increasing amounts of evidence indicate that OCD and related disorders have some radical differences from anxious disorders, including feedback processing [33]. In fact several studies revealed that anxiety can in a large extent impact on processing of external feedback. Using a simple gambling task, Gu et al found that high trait-anxiety individuals showed smaller FRN amplitude compare with low trait anxiety, which indicated there is a relationship between FRN and anxiety [34]. There are some differences of mental characteristics, cognitive processing, and neural activity for feedback processing between anxiety and obsessive-compulsive (OC).

Firstly, the difference in FRN pattern can be related to different style of locus of control (LOC). High anxious individuals have a more external locus of control (LOC), whereas the OC individuals have a less external LOC [35]. The LOC has been reported to evaluate the attribution style which refers to the tendency to ascribe the cause of actions or events to either internal or external drives or forces, a larger FRN for individuals is associated with a more internal LOC [36]. Higher levels of trait anxiety individuals showed a lower FRN as a result of the weaker link between internal LOC and FRN [34]. For OC individuals, they tend to attribute the negative results of self reasons rather than the outside ones. Therefore, they may have higher FRN in response to negative feedback results. Secondly, the OC individuals have higher needs for control than other anxious patients [37]. In decision-making tasks under ambiguity, senses of control would have been challenged, which may affect the emotion and influence the processing of negative feedback results. Thirdly, trait anxiety is more likely to impact the prediction of negative results during the processing of action-outcome sequences. High anxious individuals would hold more negative outcome expectations [38], [39] and there would be a diminished discrepancy between the actual and expected outcome under negative external feedback, as a result of which, high anxious individuals would show blunted FRN to negative feedback. In contrast, perfectionism and the avoidance of mistakes [40] would let OC individuals have higher expectations on results, which could lead to a higher discrepancy between the actual and expected outcome, and this might increase the FRN under negative external feedback. Lastly, OCD shows its difference from other anxiety disorders concerning fronto-striatal circuitry (including OFC, ACC, and striatum) [41], which is known to function in error detection and implicit learning. Furthermore, evidences from voxel-based morphometry studies suggested that compared to other anxiety disorders, individuals with OC have increased gray matter volumes in bilateral neostriatum which were associated with error processing. In fact, the DSM-5 chapter on anxiety disorder no longer includes obsessive-compulsive disorder. To this end, it is essential to clarify the potential factors that cause these confused and conflict results. Because of the relatively low FRN amplitudes indicating negative versus positive outcomes of the high trait-anxiety individuals, we cannot determine whether the altered FRN was caused by anxiety or the obsessive-compulsive [34]. Therefore, it is necessary to study the FRN characteristics in OC individuals and eliminate the interference of anxiety symptoms from the analysis. Although it is hard to solely verify the effect of OC symptoms on error processing and monitoring without the obstructions of anxiety in clinical OCD patients, it is absolutely feasible to overcome this issue by studying specific subclinical OC individuals that have not displayed obvious anxiety symptoms.

FRN is usually evident in tasks in which precise predictions are impossible, and feedback is closely related to economic benefit. Some neuropsychological research in OCD patients has found impaired decision-making behavior on the Iowa Gambling Task (IGT) [42], [43]. IGT is a widely used instrument to assess decision-making under uncertainty [44]. Gehring and Willoughby (2002) firstly reported that the FRN became larger after losses than after gains in gambling tasks. The gambling task has been used as an appropriate and classic paradigm to investigate alterations in the FRN and FRN-related brain activities in negative feedback processing in OCD [10], [11], [23], [34]. FRN was reported to robustly occur during slightly negative outcomes [19]. In the current study, we developed a modified IGT to observe external error feedback processing in SOC subjects.

Therefore, the purpose of the present study was to examine the FRN following negative feedback stimuli in SOC subjects without obvious anxious symptoms under a gambling paradigm. The hypothesized result of increased FRN amplitude would further support the assumption that increased external error processing occurs in SOC. To understand the possible source of FRN differences between SOC and comparison subjects better, we used source localization methods during the FRN time window. These results may further elucidate the electrophysiological and neural basis of decision-making deficits in OCD.

## Materials and Methods

### Subjects

Undergraduate students (628, all Chinese) from Anhui Medical University completed the Chinese Version of the Padua Inventory-Washington State University Revision (PI-WSUR) [45]. The PI-WSUR is intended to be used for screening as well as evaluating symptom severity of OCD [46]. The inventory has been tested on Italian, American, Dutch, and Australian samples [47]–[50]and has excellent psychometric proprieties. The Chinese version also has high internal consistency and good test-retest reliability [45]. We chose fourteen students (7 women, 7 men) who scored in the upper 10% of the distribution and fourteen students (5 women, 9 men) who scored in the lower 10% of the distribution as the subclinical obsessive-compulsive subjects (SOC) and low obsessive-compulsive group (LOC), respectively. All of the subjects agreed to participate in the study. They were screened to exclude the possibility that they met the clinical criteria for OCD, substance abuse, neurological diseases, or any other psychiatric diseases according to the Tenth Edition of the International Classification of Diseases (ICD-10). In particular, the subjects exhibited no anxiety or depressive symptoms (Hamilton Anxiety Rating Scale [HAMA-14] scores≤14 and Hamilton Depression Rating Scale [HAMD-17] scores≤17). There were no significant differences between the 2 groups in sex, age, handedness, HAMA, HAMD and PI-WSUR scores (Table 1). The study was approved by The Ethics Committee of Anhui Medical University. All of the participants gave written consent and received a monetary reward.

**Table 1:** Group characteristics of the SOC group and the LOC group.

	SOC (N = 14)	LOC (N = 14)	Between-groups comparison
	Mean (SD)	Mean (SD)	P-value
Sex (males/female)	7/7	9/5	0.45
Age (years)	19.86(1.07)	19.64(1.15)	0.70
Handedness (R/L)	14/0	13/1	0.32
HAMA	4.67(4.16)	2.17(1.64)	0.07
HAMD	4.08(3.98)	2.08(1.88)	0.14
PI-WSUR score (total)	49.17(15.86)	6.75(4.85)	<0.001

Abbreviations: SOC, subclinical obsessive-compulsive; LOC, low obsessive-compulsive; HAMA, Hamilton Anxiety Rating Scale;HAMD,Hamilton Depression Rating Scale;PI-WSUR,Padua Inventory-Washington State University Revision.

### Stimuli and Experimental Procedure

**Figure 1:**
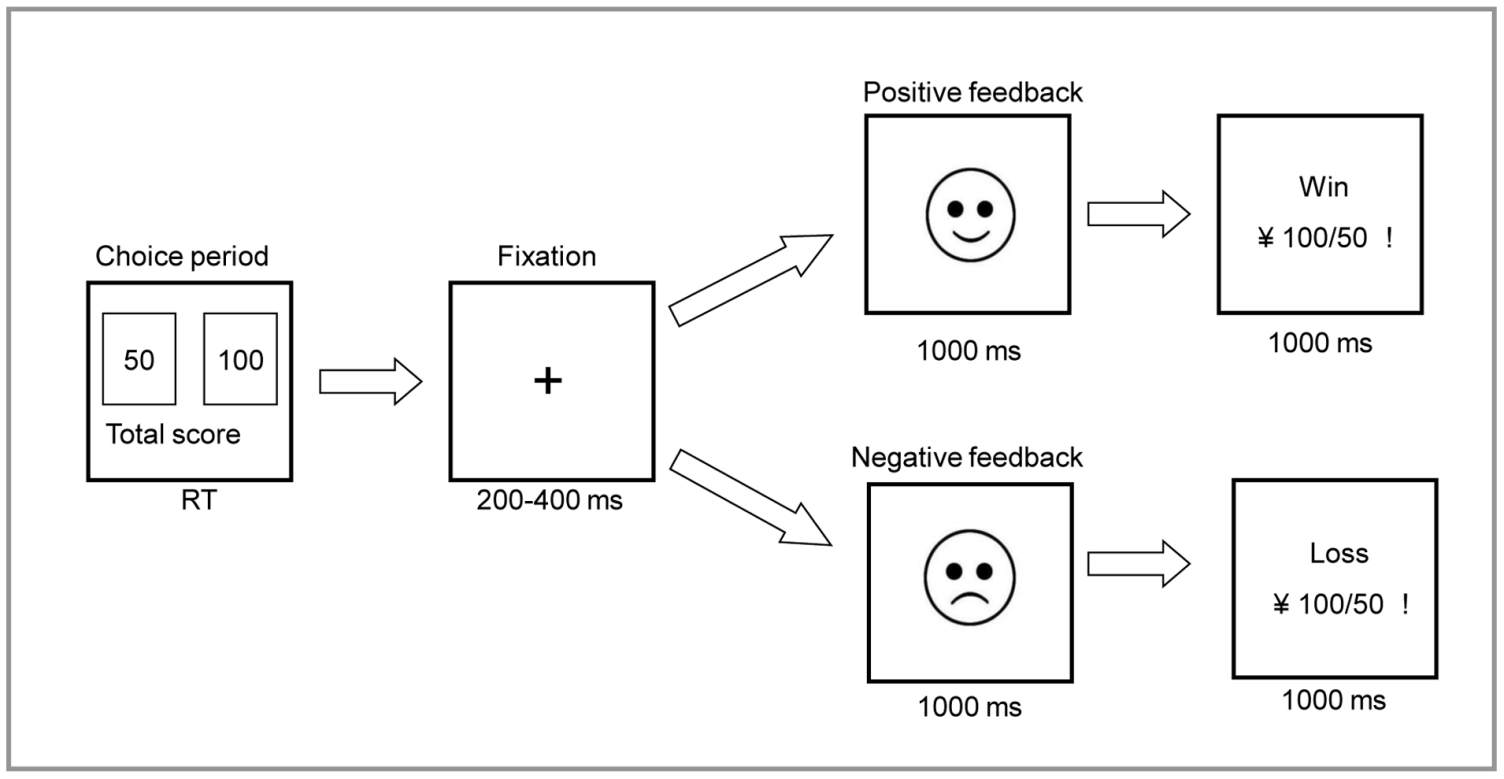
The presentation sequence within a single trial of the revised Iowa Gambling Task (IGT). On each trial, the participants were presented with a choice of two alternatives, one of which they were asked to select using their left or right index finger. The presentation would remain until the button press. After a fixation point appeared and lasted for 200–400 ms, the participants received feedback from a cartoon face for 1000 ms, indicating whether they lost or won in the trial. Subsequently, a numerical stimulus popped up on the computer screen to indicate the selected consequence, which lasted for 1000 ms (RT: response time).

The subjects performed a gambling game related to the Iowa Gambling Task (IGT) (Fig. 1) [44], a laboratory task specifically developed to measure decision-making based on initially implicit probabilities. At the start of each trial, a choice stimulus (CS) with two numbers, a 50-point bet (left box) and a 100-point bet (right box), represented the monetary value in RMB. Each bet was associated with a defined win/loss ratio as well as different winning probabilities: a 0.6/0.4 win/loss probability for the 50-point bet and a 0.4/0.6 win/loss probability for the 100-point bet. But the sequence of negative and positive feedbacks is random. Thus, the 50-point bet was a advantageous choice, and the 100 point bet was an disadvantageous choice. However, the subjects were not made aware of the loss/gain probability or the sequence of the task over the experiment. After selecting a bet, the display went blank except for a central fixation cross, which lasted for 200 ms to 400 ms. A cartoon face (the outcome stimulus, OS) then appeared to indicate whether the bet was a win (a smiling face) or a loss (a depressed face). The face was present for 1000 ms. Then, a numerical stimulus (NS) indicated the amount either in the win or loss condition. This numerical stimulus remained visible for 1000 ms. The choice stimulus reappeared to initiate the next trial. The task was divided into three segments with each 100-trial segment lasting for approximately 4 min. The procedure was identical in each block. At the end of each segment, the overall “loss” or “win” status over the entire block was displayed on the monitor screen. The subjects were instructed to win as much money as possible with a starting capital (¥1000). Thus, in this task, negative feedback occurs after making a choice error.

### Electrophysiological Recordings

Electroencephalogram (EEG) data were measured from 64 scalp sites using Ag/AgCl electrodes mounted on an elastic cap (NeuroScan, Sterling, Virginia, USA) and positioned according to the international 10/20 system. A forehead electrode was used as the ground. All EEG channels were referenced to the left mastoid. Electrooculograms (EOG) were recorded bipolarly both horizontally from the left versus right orbital rim and vertically from a pair of electrodes supraorbital and infraorbital to the left eye. All electrode impedances were maintained below 5КΩ. The EEG and EOG activity was amplified with a 0.01–100 Hz band pass filter and continuously sampled with the 500 Hz/channel. The acquired signals were stored for subsequent analyses.

Ocular artifacts were removed from the EEG signal using a regression procedure implemented in the used Neuroscan software [51]. The EEG data were off-line re-referenced to the average of the left and right mastoids and digitally low-pass filtered below 30 Hz. Any trials with a signal exceeding ±100 µV were excluded from averaging to eliminate EOG and movement artifacts. The ERP waveforms were cut from 200 ms before the onset of the feedback cartoon face to 1000 ms after (with 200 ms pre-stimulus as baseline). The FRN time windows were established from grand averages (group collapsed) based on at least 40 trials. Converging previous ERP studies reported FRN reached the largest amplitude at frontocentral midline sites [52], [53], [54], [55]. Some studies quantified FRN amplitude at the single electrode, such as at Fz [21], FCz [14] and Cz [56] where the FRN amplitude were largest. To avoid on chance where FRN is maximal, we selected Fz, FCz, Cz and CPz as a small cluster to quantified the FRN amplitudes. We measured the FRN time window in 240–340 ms based on the peak of FRN from electrode FCz, where this ERP component typically reached maximum amplitude and occurred at 290 ms. The average amplitude measure was used because it could weaken the noise fluctuation compare with base to peak approach in ERP waveform, which is insensitive to positive deflection in FRN time window [57], [58].

### sLORETA Source Analysis

Standardized low-resolution brain electromagnetic tomography (sLORETA) was used to estimate the cerebral generator underlying the FRN (240 ms to 340 ms) [59]. The sLORETA technique provides a three-dimensional discrete linear solution with zero localization error and has been frequently used for EEG source analysis. This method estimates neuronal activity as current source density (CSD) restricted to the cortical grey matter and the hippocampus using a digitized MNI atlas with 6239 voxels at a spatial resolution of 5 mm. In general, the validation of the sLORETA method has been independently replicated and cross-validated with fMRI and other brain imaging methods [60]–[63].

To identify the different neural responses to negative feedback between the high and low group, we compared voxel-based whole-brain sLORETA images between the groups during the loss condition based on the statistical non-parametric mapping (SnPM) methodology [64]. In addition, a region-of-interest (ROI) approach was performed to explore the CSD of the regions detected in the first step. As reported by previous studies, the dACC was involved in error monitoring and cognitive control processes [22], [65], the ROI for the dACC were submitted for further CSD analysis [66]. The ROIs (radius = 5 mm) for the brain areas detected in the first step were defined based on the coordinates of the local peak activation voxel obtained during the first pass, whereas the ROI for the dACC (BA32, x = 4, y = 18, z = 44) were determined based on previous literature.

### Statistical Analysis

All behavioral and electrophysiological analyses were conducted using the SPSS software package (Version 16.0; SPSS Inc., Chicago,USA). To analyze the task behavioral performance, we calculated the total netscore by subtracting the number of disadvantageous choices (100 point bet) from the number of advantageous choices (50 point bet). The 300 trials were divided into 6 equal blocks, and the netscore of each block of 50 choices was calculated to investigate whether decision making changed during the task. Repeated-measures ANOVAs were performed on the differences in value between the beneficial and adverse selections using blocks as within-subjects factors and group as the between-subjects factor. The average amplitudes were submitted to multivariate repeated-measures ANOVAs with feedback type (loss and win), intensity (50 as low condition and 100 as high condition) and electrode (Fz, FCz, Cz, CPz) as within-subject factors and group (SOC subjects and LOC subjects) as the between-subject factor. The degrees of freedom of the F-ratios were adjusted according to the Greenhouse-Geisser (GG) epsilon correction in all analyses. In addition, the nonparametric Spearman correlations between the average amplitudes during the loss condition and the score of PI-WSUR in the SOC subjects were calculated to see if the average amplitude of FRN was selectively related to OC symptoms. In addition, we calculated the correlations between clinical behavioral performance and ERP measures. Moreover, a correlation analysis on the CSD was also conducted between dACC and brain areas detected by source analysis to assess whether those brain areas were functionally connected.

## Results

### Behavioral Results

**Figure 2:**
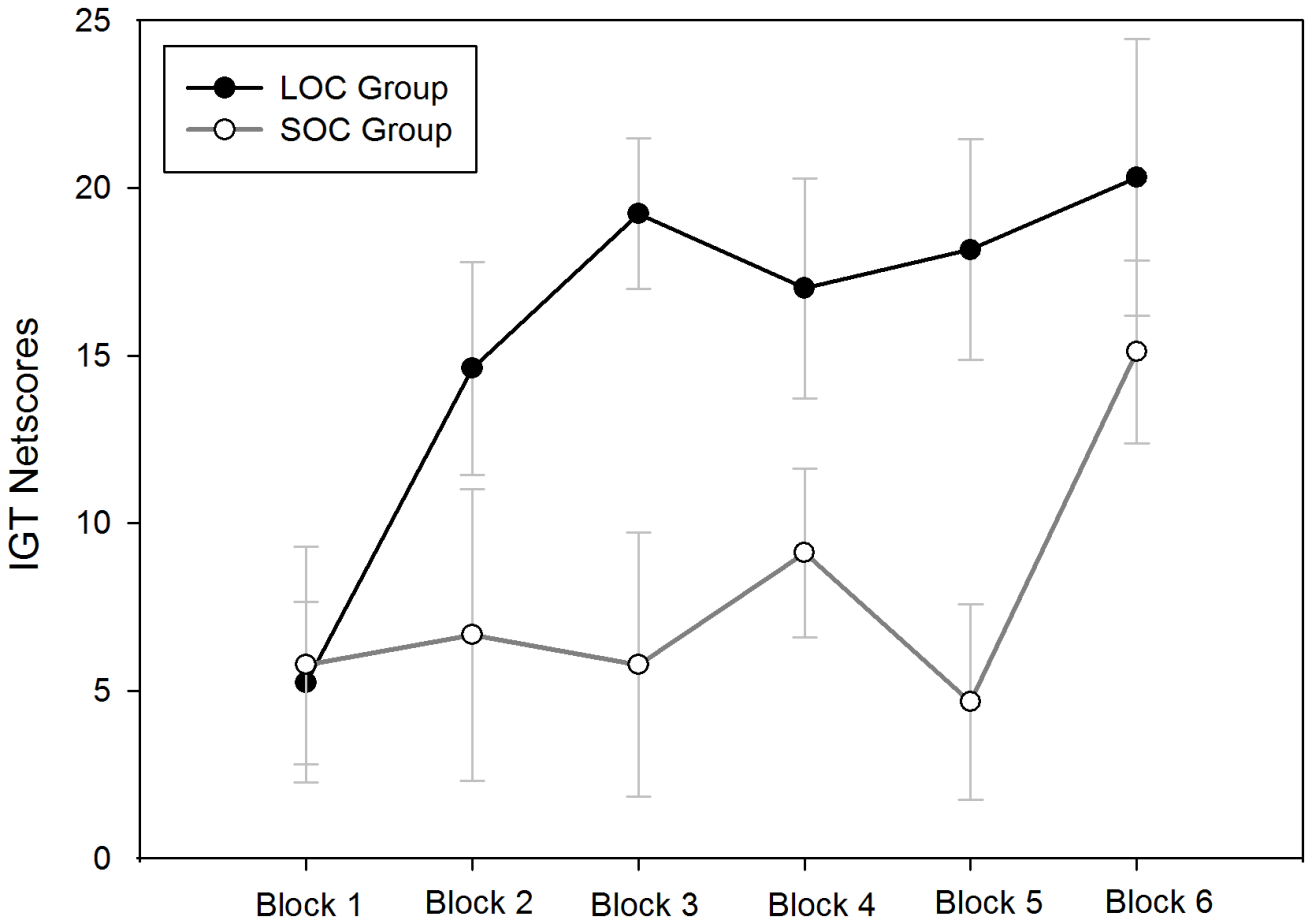
Performance of SOC and LOC in the revised Iowa Gambling Task (IGT). Means of the net scores (the number of disadvantageous choices minus the number of advantageous choices) and standard error are presented for the six IGT blocks (block 1 = trials 1–50, block 2 = trials 51–100, block 3 = trials 101–150, block 4 = trials 151–200, block 5 = trials 201–250, block 6 = trials 251–300) (SOC: subclinical obsessive-compulsive; LOC: low obsessive-compulsive).

The repeated-measures ANOVA of the netscores revealed significant effects for the blocks factor [*F*(5,130) = 2.95, *P*<0.05] and a block by group interaction effect [*F*(5,130) = 2.77, *P* = 0.05]. The simple analysis revealed that no significant main effect for blocks were detected in the SOC subjects [*F*(5,78) = 1.82, *P* = 0.12], indicating that the SOC subjects selected from the disadvantageous choices with a higher frequency in the IGT task. However, there was a significant main effect for blocks in the LOC group [*F*(5,78) = 3.062, *P* = 0.015]. According to the LSD tests, the netscores of the LOC group between block 2 to block 6 were significantly higher compared to block 1 (all *P*’s<0.05). The net score of the LOC group markedly increased over the task, indicating that the increased advantageous choices (learning main effects) in the LOC group was obvious (Fig. 2).

### ERP Results

**Figure 3:**
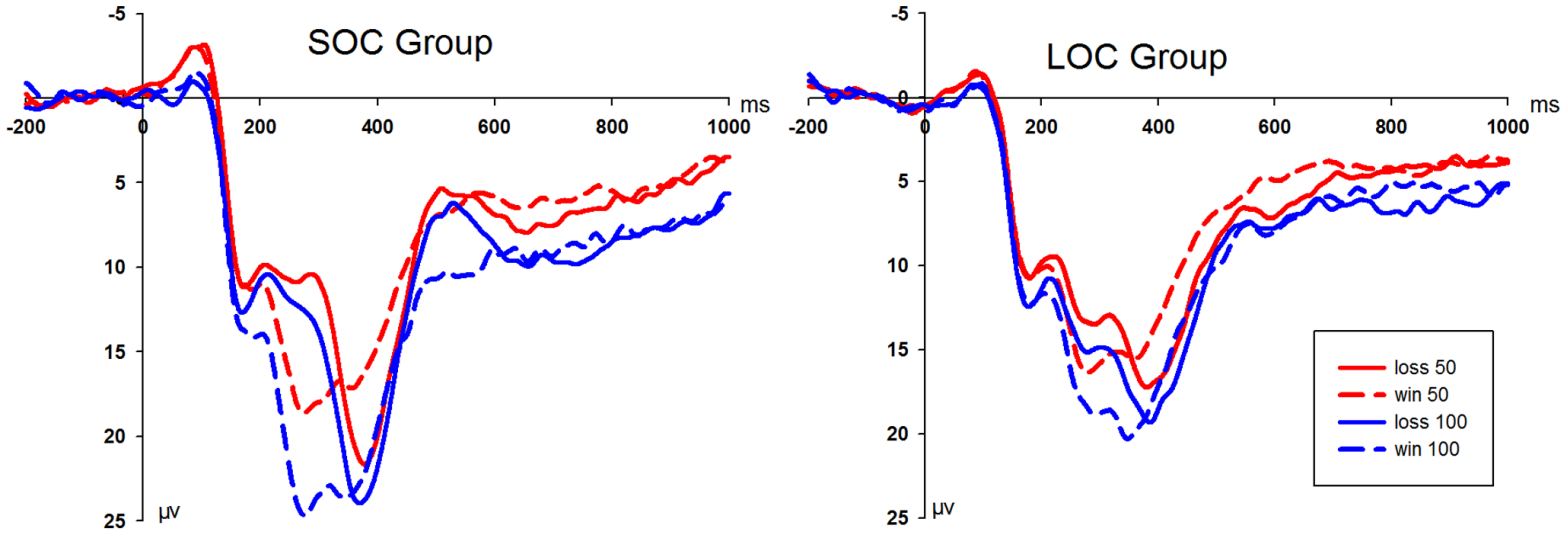
Grand averages evoked by the feedback face at FCz in two groups. Grand averages evoked by the loss (solid lines) and win (dash lines) feedback face under the low (red lines) and high conditions (blue lines) at FCz recording sites in the SOC (left panel) and LOC (right panel) groups (SOC: subclinical obsessive-compulsive; LOC: low obsessive-compulsive).

The repeated-measures ANOVA of the average amplitude of the original FRN waveform revealed significant main effects of feedback type [*F*(1,26) = 76.35, *P*<0.001], intensity [*F*(1,26) = 18.70, *P*<0.001] and electrode [*F*(3,78) = 29.25, *P*<0.001]. The largest FRN amplitude at the FCz site (12.74±1.17 µV) was more pronounced during the loss (13.33±1.13 µV) versus win (17.63±1.24 µV) conditions. The amplitude was larger in the low (14.15±1.14 µV) versus high loss conditions (16.81±1.26 µV) (Fig. 3). More importantly, the interaction effect of feedback type and group was significant [*F*(1,26) = 15.42, *P*<0.01]. This simple analysis of effects revealed that the amplitude differences between the loss and win conditions were larger in the SOC group compared with the LOC group.

**Figure 4:**
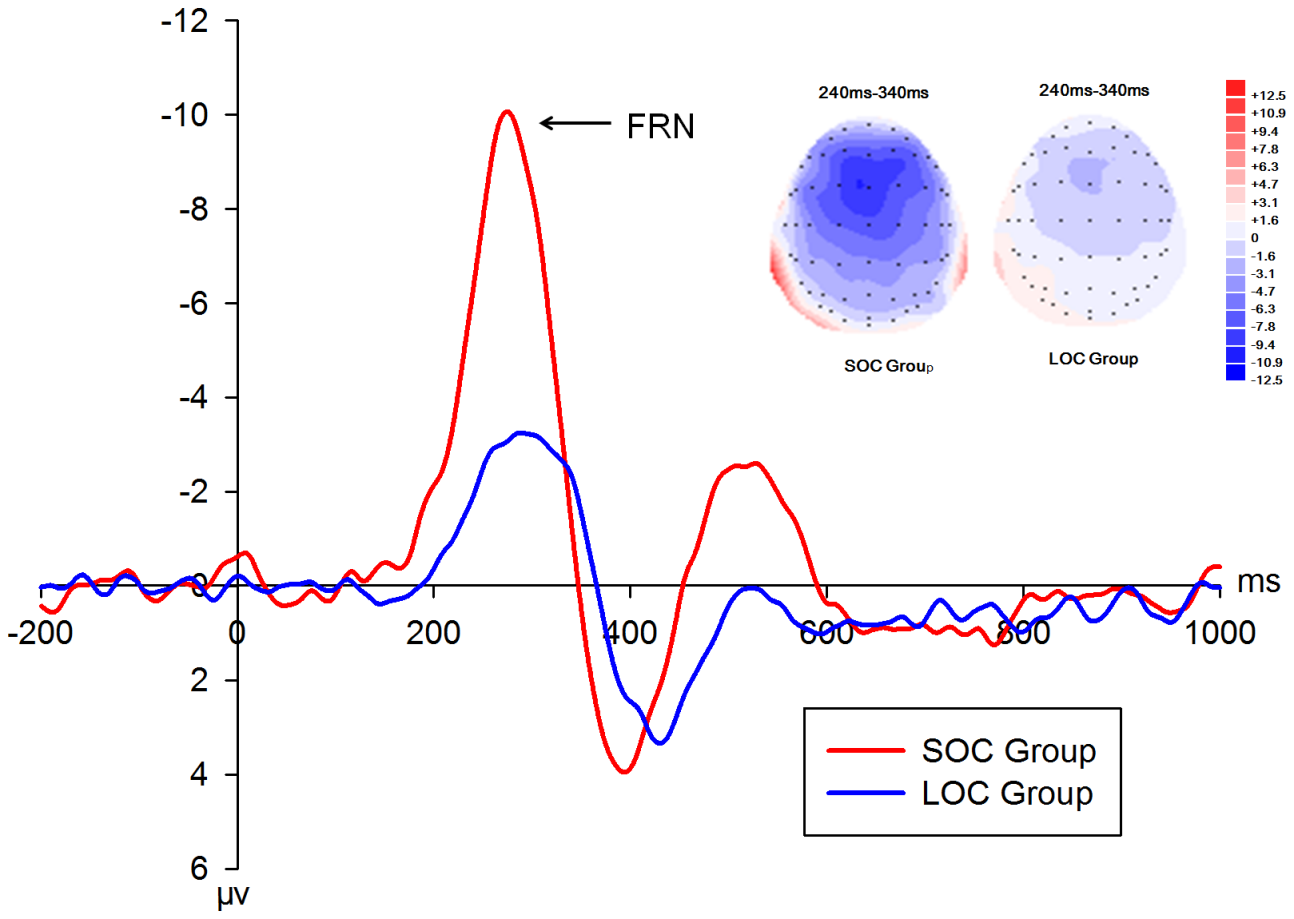
Feedback-related negativity (FRN) difference waves and corresponding scalp topographies of the two groups. FRN Difference waves (loss-win) of the SOC and LOC groups at FCz (left panel), as well as the corresponding scalp topographies (right panel). The shaded area indicates the 240–340 ms time window in which the FRN was analyzed (SOC: subclinical obsessive-compulsive; LOC: low obsessive-compulsive).

To clearly illustrate the interaction effect between feedback type and group, the amplitude of the loss-win difference waveform was analyzed. A repeated-measures ANOVA on the amplitude of the FRN difference wave revealed significant effects of electrode [*F*(3,78) = 25.28, *P*<0.001], intensity [*F*(1,26) = 6.56, *P*<0.05], and group [*F*(1,13) = 62.47, *P*<0.01]. The FRN amplitude, mainly distributed in the FCz site (−6.14±0.66 µV), was larger in the high (−8.93±1.05 µV) versus low loss condition (−6.27±1.14 µV). The SOC group (−7.60±4.57 µV) displayed larger amplitude than the LOC group (−2.59±3.01 µV) (Fig. 4).

### Relationship between Clinical Characteristics and Task-Related Measures

Significant correlations were not found between the clinical measures (PI-WSUR) and the netscores in behavioral results, which also occurred in the relationship of clinical measures and average FRN amplitudes under loss conditions. We observed that FRN is negatively correlated with netscores in behavioral results in SOC group. This suggested that in the SOC group the greater amplitudes of FRN are related to the worse behavior performance. To further clarify whether FRN group differences of negative feedback were independent of anxious symptoms, the HAMA score was entered as a covariate in an analysis of covariance. The interaction effect of feedback type and group for FRN amplitudes was still significant (*F*(1,26) = 6.811, *P*<0.05), indicating that anxiety did not affect the analyses of behavior and FRN results on group differences.

### Source Localization Results

**Figure 5:**
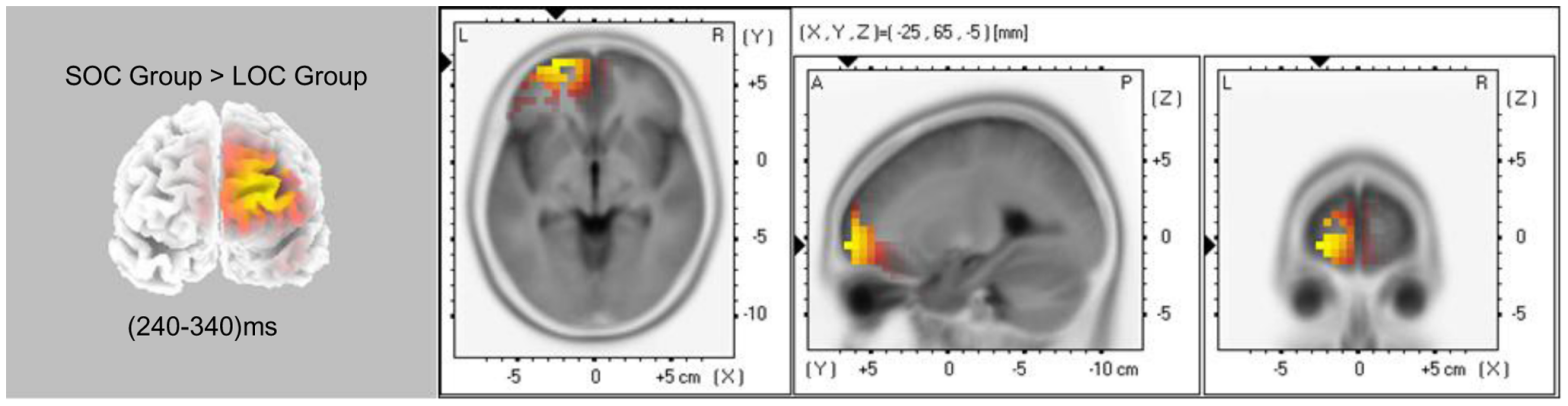
sLORETA solutions to the non-parametric randomization tests on the FRN component in the loss condition. Standardized low-resolution brain electromagnetic tomography (sLORETA) solutions to the non-parametric randomization tests on the FRN component in the loss condition showing voxels in which the SOC>LOC contrast was significant (*P*<0.05) (SOC: subclinical obsessive-compulsive; LOC: low obsessive-compulsive).

As hypothesized, the brain regions involved in negative feedback processing varied between the SOC and LOC groups, indicating functional abnormality in the SOC subjects. The current source density in the anterior prefrontal cortex (aPFC), the intersection between the left frontal pole and the left orbito-frontal cortex (BA10/11), was significantly greater in the SOC group compared with the LOC group (*t* (26) = 4.68, *P*<0.05) (Fig. 5). The current source density in the dACC was marginally greater in the SOC group compared with the LOC group, but it did not reach statistical significance (in low loss: t (26) = 1.84, P = 0.08; in high loss: t (26) = 1.08,P = 0.29).

**Figure 6:**
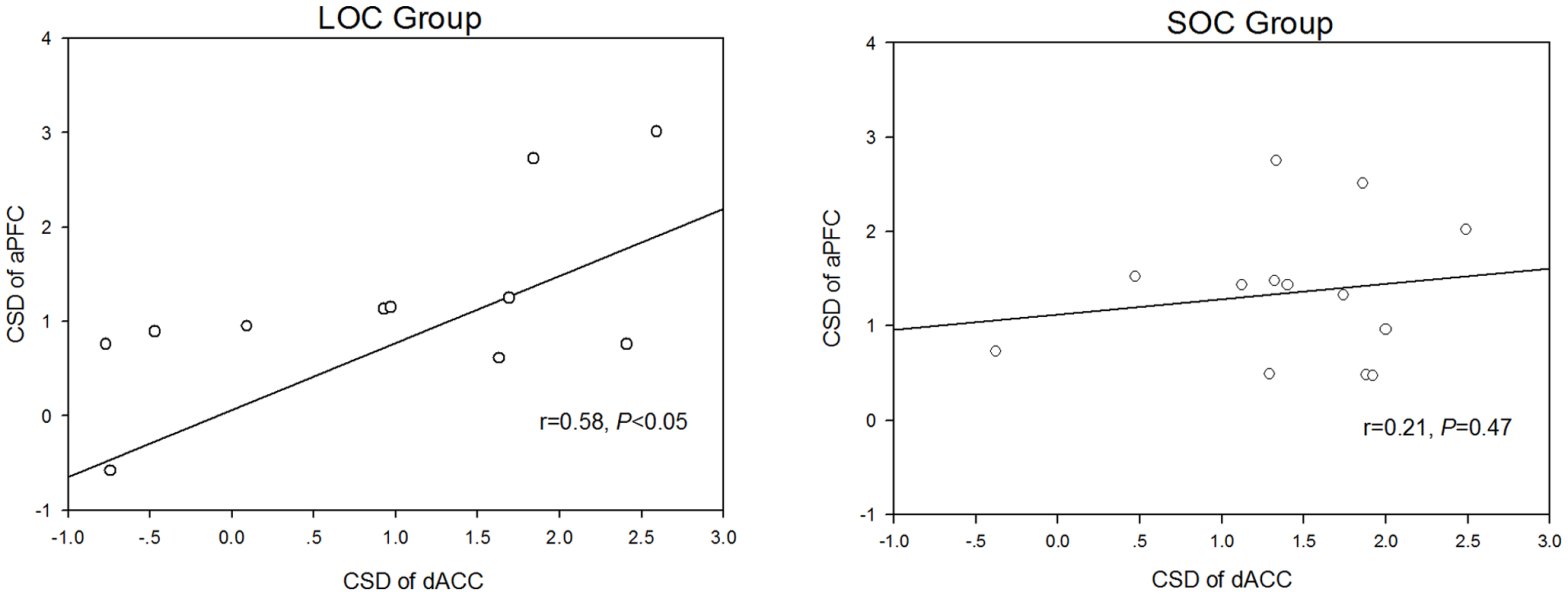
Scatter plots of aPFC and dACC activation during the FRN time window. Scatter plots of anterior prefrontal cortex (aPFC) and dorsal anterior cingulate cortex (dACC) activation during the FRN time window in the SOC (right panel) and LOC (left panel) groups (CSD: current source density; SOC: subclinical obsessive-compulsive; LOC: low obsessive-compulsive).

Furthermore, a correlation analysis of the dACC and aPFC indicated a disassociation between the LOC group and the SOC group (Fig. 6). In the LOC group, the current density in the dACC was significantly correlated with that in the aPFC (r = 0.58, *P*<0.05), whereas the analysis failed to reveal a significant correlation in the SOC subjects (r = 0.21, *P* = 0.47).

## Discussion

In the present study, we examined behavioral performance and the spatial-temporal features of external negative feedback processing using a modified IGT task in SOC subjects without apparent anxiety. As expected, the observation of a general feedback-related negative wave effect for the loss and win conditions (loss>win) replicated the results of many previous studies [19], [21], [67]. Convergent electrophysiological studies on external negative feedback processing suggest that, as identified in gambling tasks, the FRN reflects reward prediction errors in the feedback monitoring system. This type of error could trigger negative affect responses to monetary loss, or alternatively, negative affect could signal the need to adjust behavior [9], [19], [68]. In the present study, the subjects were instructed to win as much money as possible and losing money was known an error and constituted an unexpected negative outcome. As more money was lost, greater deviation in expectancy was produced. Thus, we can understand the FRN effect of feedback type in all participants.

More importantly, the SOC subjects displayed enhanced amplitude of the original FRN waveform differences between the loss and win conditions when compared to the non-compulsive LOC subjects. Previously, it has been demonstrated that the ERN is greater for unexpected error outcomes in OC groups than healthy individuals [12], [27], [69]. However, the ERN findings differed from the results of FRN amplitudes in subclinical populations with OC symptoms and OCD, in which reduced FRN has been discovered [30]–[32], [70]. The explanation of these results may relate to the bias for overestimation of possible negative outcomes in OC populations [71], [72], but such an explanation does not rule out the influence of anxiety. In fact, higher levels of anxiety are associated with the expectancy of a more negative outcome in decision-making task [73], and therefore the reduced FRN detected in previous studies was likely related to the effects of anxiety. All the studies we mentioned above did not measure anxiety symptoms using scales or any other approaches. Although OCD patients often display anxiety symptoms, anxiety is not the core manifestation of obsessive-compulsive disorders [33]. Differentiated from anxiety disorder, several studies have found a significant relationship between perfectionism and obsessive-compulsive symptoms in non-clinical populations, and the major feature of perfectionism is the avoidance of mistakes [76]. Therefore, the expectancy of outcome may be more positive. The difference in FRN pattern can also be related to different style of LOC. High OC individuals have a more internal LOC [35] which is associated with a larger FRN. Ambiguity in our gambling task may affect the emotion and the FRN results. Furthermore, OCD may be distinguished from other anxiety disorders in that the hyperactivity and hyperresponse within the OFC, ACC, and caudate are not found in other anxiety disorders according to neurocircuitry findings [74], [75]. To study the relationship between OC and FRN, we need to exclude the influence of the anxiety. In our study, we chose the SOC participants whose anxiety score did not reach the diagnosis standard, and our ERP results were also consistent with the suggestion of larger FRN in OCD patients [29], and the larger amplitude differences in the SOC reflect the excessive monitoring to error feedback. Covariance analysis revealed that anxiety has no obvious impacts on the FRN results. This is the first finding that proves the difference between obsessive-compulsive and anxiety traits in the context of neural electrophysiology.

As expected, our study revealed the SOC subjects were impaired in their decision making and selected from the disadvantageous choices with a higher frequency in the IGT task compared with LOC subjects. The behavioral deficits that occurred in the SOC group are also supported by some IGT studies in OCD patients [42], [43]. The reinforcement learning theory states that the FRN indexes an evaluative signal that depends on outcome valence and outcome expectancy whereby larger FRN precede behavioral adjustments [76]. The current study demonstrated increased FRN for loss feedback in the LOC group and a positive correlation with the behavior adjustment toward a favorable direction, which are both consistent with the reinforcement learning theory. However, a larger FRN did not prompt the behavioral performances in SOC group, which suggests that an appropriate degree of error feedback monitoring was helpful for guiding behavioral adjustments in terms of outcome errors or violations of expectations about those outcomes [18]. A theoretical inverted-U model of cortical dopamine (DA) function can be used to explain the conflict effect of FRN on behavioral adjustments in the context of reinforcement learning models. It has been suggested that both insufficient and excessive DA-receptor stimulation lead to poor performance on DA-dependent tasks such as gambling tasks [77]. Several sources of evidence suggest that the FRN is moderated by dopaminergic function [10]. Therefore, it is plausible to infer that the contradiction between the behavioral and electrophysiological results reflect an inappropriate level of cortical dopamine in the SOC group. Similar to the ERN measured in a previous study [78], no significant correlation was obtained between FRN amplitudes and symptom scale scores. We can infer that overactive external error monitoring may reflect a endophenotype which is closer to the underlying neuropathology than top-level clinical symptoms for SOC. Thus, the obtained results support the idea that enhanced FRN represents a candidate neurocognitive endophenotype of OCD.

Consistent with other studies [74], [79], our current work also documented that SOC subjects displayed more aPFC (the joint part of the frontal pole cortex and the OFC, BA10/11) hyperactivation compared with LOC subjects under the loss condition, a finding revealed using sLORETA during the FRN time window. Considerable neuroimaging research in humans indicates that the frontal pole cortex (also known as BA10) contributes to learning goal-generating processes in a controlled and flexible fashion and improves future choices in decision-making conditions that produce evident costs and benefits. Such a protracted function could be advantageous for the adaptation to complex social and cultural environments. However, in some developmental abnormalities such as OCD, excessive activation in the frontal pole cortex is likely to be related to opposite functions [80]. Convergent reports have emphasized the key role of the OFC in the pathophysiology of OCD [81], [82]. Recent research also implies a function of the OFC in processing feedback valence [83], [84], representing punishing outcomes, identifying bad information and escaping from danger [85], [86], suggesting that the OFC may be involved in ritualized behavioral responses. Our results revealed that an overactive representation of negative feedback information and a decreased ability to learn from changing representations may be important cognitive and biological mechanisms of OC individuals.

Because dACC hyperactivation during FRN and the sensitivity of dACC to external sources of error information have been confirmed in some previous studies, we explored the correlation between the dACC and the aPFC in two groups to clarify how these two areas work together in negative feedback processing [9], [10], [19]. However, we did not find any correlation in current density between the dACC and the aPFC in SOC subjects, a relationship that certainly exists in LOC control subjects during the loss condition. This suggests that there may be dysfunctions in the aPFC-dACC frontal network in SOC subjects that underlie impaired decision-making abilities. In negative feedback processing, the dACC is responsible for monitoring and integrating the representation of negative feedback stimuli and the reinforcement history, ultimately guiding the next action choice [18], [57]. It appears that increased activity in the aPFC and reduced connectivity between the aPFC and dACC are associated with hypersensitivity to the negative characteristics of a stimulus derived from negative feedback and a reduced adjustment to aPFC hyperactivation. This illustrates a dysfunction in the interaction between aPFC and dACC during negative feedback processing in SOC subjects. These results further enrich the understanding on neural mechanism of OC underlying decision making dysfunction. Future investigations are needed to replicate these results with an optimized methodology (such as PET or fMRI) for functional localization with large samples.

## Conclusions

Taken together, our results demonstrate the difference between SOC subjects without obvious anxiety and LOC controls in FRN. SOC subjects displayed greater amplitudes of FRN during the presentation of loss feedback and greater FRN amplitude differences between loss and win conditions compared with LOC controls. Significant correlations were not found between clinical measures and the FRN amplitudes under loss conditions. We also found SOC subjects were impaired in their decision making compared with LOC controls. Importantly, our results confirmed that SOC subjects display larger aPFC activation in response to negative feedback stimuli and a dysfunction in the interaction between aPFC and dACC during FRN production in SOC. These results indicate that interventions to adjust the cognition to negative feedback and strengthen interconnectivity between the dACC and aPFC may improve decision making functions in OC-related individuals.
